# Medical and Philosophical News

**Published:** 1781-08

**Authors:** 


### SECTION III.

### Medical and Philosophical News.

THE Royal Academy of surgery at Paris have proposed the following question for a prize of twelve hundred livres: "What is "the best method of removing with safety those "blemishes in the face that are occasioned by "gunpowder?"—The dissertations on this subject are to be written in French or Latin, and sent to M. Louis, secretary to the academy, before the 1st of April 1782.

---

Extract of a letter from Professor Ploucquet, of the university of Tubingen, to a member of the society:

"Some time ago I had occasion to treat a case of inguinal hernia in a female patient. It appeared in the right groin, but was so small that it was impossible to determine whether it was a portion of intestine or omentum that protruded. The patient was troubled with colic, vomiting, and the other usual symptoms of an incarcerated hernia. Repeated attempts were made to reduce it, but without effect, as it constantly

ſtantly ſlipped from under my fingers, till at length upon introducing two of my fingers into the vagina, and placing them immediately under the hernia, while with my other hand the neceſſary preſſure was made externally on the abdomen, I eaſily ſucceeded in the reduction. It has returned ſeveral times ſince, but has always been reduced in the ſame manner without the leaſt difficulty. Perhaps a ſimilar method might be uſeful in caſes of this ſort in men. For this purpoſe the two fingers might be introduced into the anus."

---

By a letter from Sweden we are informed, that a ſaturated infuſion of *Ledum paluſtre*, continued for three or four months in leproſy, has effected a cure in ſeveral caſes without the aſſiſtance of any other remedy.

---

Extract of a letter from Dr. Michaelis, phyſician to the Heſſian troops in America, to Profeſſor Richter at Gottingen:

"It is remarkable, that patients labouring under a certain degree of ſcurvy cannot bear the open air. Several ſcorbutic patients on board board our ſhip, when upon deck, intreated me for God's ſake to order them to be carried down to that part of the veſſel where they were accuſtomed to paſs the night, and where the air was very bad, on account of the great number of people collected there night and day. Many of them died ſoon after their landing; as did a great number of other ſcorbutic patients belonging to the fleet. It is well known here, that of the ſcorbutic people who have long been confined in a ſhip's hold, many die as ſoon as they are brought upon deck, others in the boat that conveys them aſhore, and ſome ſoon after their landing. We can hardly aſcribe the deaths of ſuch perſons to fatigue (which indeed, when in a certain degree, might be very fatal to patients in ſo reduced a ſtate) for they are carried upon deck in their beds, and conveyed from thence in the ſame manner into the boats. The fact is, that the pure air is too elaſtic for their enfeebled organs of reſpiration, ſo they die ſuffocated."

"From the experiments made here by the Engliſh army phyſicians Columbo root appears to have great powers in hypochondriacal affection, and to be an excellent medicine in the dyſentery, provided the neceſſary evacuations have been been premiſed. They have likewiſe found the Arnica root of uſe in caſes where the principal indication was to ſtrengthen."

---

Dr. Sigault, phyſician at Paris, has lately experienced good effects from the external uſe of cold water, in a caſe where the progreſs of labour was retarded by violent convulſions. Upon throwing a pailful of water (in which ice had been previouſly diſſolved) on the patient, the ſpaſms inſtantly ſubſided, and the os uteri ſoon began to dilate. By a few repetitions of this application the convulſions were entirely removed, and the os uteri in a ſhort time was ſufficiently open to allow of the introduction of the forceps, with which the patient was delivered of a dead child.

---

The following account of the Cæſarean operation performed lately at Lyons is extracted from a periodical work, entitled " Obſervations " ſur les maladies regnantes à Lyon, par M. M. " Vitet et Petetin, medecins:"

" Pierette Mornon, a delicate woman aged 27 years, much deformed by the rickets, was brought to the hoſpital, February 25, 1781, in labour. At ten o'clock in the forenoon the os uteri was dilated to the ſize of a half crown. At eleven, the waters being evacuated, the head of the fœtus was felt above the brim of the pelvis, which it was prevented from entering by the too great projection of the os ſacrum, where it joins the laſt lumbar vertebra. It being reſolved to perform the Cæſarean ſection, an inciſion was made through the integuments in the direction of the linea alba ſix inches and a half in length. The inciſion into the uterus, four fingers breadth in length, was attended with conſiderable pain and hæmorrhage. The inteſtines and omentum, having forced their way through the wound, were ſupported while the fœtus and placenta were extracted. The uterus inſtantly contracted. The inteſtines and omentum were retained by ſeveral ſtitches in the integuments, and by bandage.

" From ſix o'clock in the evening (the time the operation was performed) till midnight, the patient complained of acute pain in the wound, and her ſtrength was conſiderably diminiſhed. At half paſt one, in the morning of February the 26th, the pulſe became ſtronger and more frequent. She voided by the wound and vagina a reddiſh ſerum. At ſix o'clock the pulſe became weaker weaker and lefs frequent. At eight fhe feemed to be fomewhat relieved by an oily fomentation. At four in the afternoon fhe was attacked with vomiting, hiccup, and cold fweats, her pulfe and ftrength were funk, her extremities grew cold, her features altered, and at fix the next morning, February the 27th, fhe died.

"Upon diffection the inteftines and omentum appeared inflamed. The uterus was contracted to a moderate bulk. The diameter of the pelvis at its brim, meafuring from pubis to facrum, was about two inches; from fide to fide five inches. The iliac cavities were fmall, and bent inwards, efpecially the right.

"On dividing the fymphyfis, the feparation of the offa pubis, by the mere elafticity of the parts, left a fpace of near an inch and a half. By turning the thighs outward, and feparating them from each other, two inches were gained, and by bending them upon the abdomen the fpace was increafed to two inches and a half, by which means the diameter of the pelvis from pubis to facrum was found enlarged feven tenths of an inch, a fpace which feemed fufficient for the paffage of a fmall head, fuch as that of the dead child.

"Forty-eight hours after the diffection the mufcles of the pelvis being removed, the ligaments and cartilages that connect the facrum and ileum were examined, but did not appear to be in the leaft injured."

---

At a late monthly meeting of the College of Phyficians at Paris, M. de l'Epine communicated a cafe of fever attended with a fpontaneous hydrophobia. The patient bit a maid fervant, but without infecting her. M. Phillip fpoke of a fimilar cafe that occurred in his own practice, and proved fatal. The patient was a young lady, and the difeafe was afcribed to a fuppreffion of the menfes. M. Bajet related a cafe of tertian, each paroxyfm of which was accompanied by an eruption of broad red fpots on the fkin, which difappeared in twenty or thirty minutes.— M. Rabel mentioned a cafe of uterine hæmorrhage cured by warm bathing.

---

The following is Mr. Scheele's method of preparing the *new green colour* mentioned in our laft number:

"Two pounds of blue vitriol are to be diffolved

ſolved in ſix pints of water in a copper veſſel over the fire, after which two pounds of white, dry pot-aſh, and eleven ounces of white arſenic in powder*, are to be diſſolved in two pints of pure water in another veſſel of the ſame metal, and over the fire alſo. When this latter ſolution is complete, the lixivium is to be ſtrained through a linen bag into another veſſel.

"This arſenical lixivium is to be poured by a little at a time into the above-mentioned ſolution of blue vitriol, taking care to ſtir the mixture all the time with a wooden ſpathula †. When the whole is mixed it is to be ſuffered to reſt for ſome hours, when the green colour will fall to the bottom of the veſſel.—The clear lixivium is then to be poured off, and the hot water § repeatedly poured on the ſediment, which is afterwards to be poured on a linen cloth, and when the moiſture has filtered through it, the

* The operator will do well to powder the arſenic himſelf, as the powdered arſenic of the ſhops is often mixed with gypſum. He may convince himſelf of its purity by ſprinkling a little of the arſenic on a ſtone heated red hot. If it evaporates entirely the arſenic is pure.

† As a conſiderable effervefcence is produced by this mixture the veſſel ought to be large enough to hold two gallons.

§ As this water will contain a little arſenic care muſt be taken not to throw it into any place where animals can have acceſs to it.

green green colour is to be made into ſmall lumps and dried upon paper in a moderate heat.—The quantities above ſpecified yield eighteen ounces and a half of a fine green colour.

