# Errata

**Published:** 1781-12

**Authors:** 


					ERRATA.
Page 134, line 19, after Paris, add, aged 79 years-

				

